# How the Visitors’ Cognitive Engagement Is Driven (but Not Dictated) by the Visibility and Co-visibility of Art Exhibits

**DOI:** 10.3389/fpsyg.2020.00350

**Published:** 2020-03-03

**Authors:** Jakub Krukar, Ruth Conroy Dalton

**Affiliations:** ^1^Institute for Geoinformatics, University of Münster, Münster, Germany; ^2^School of Architecture, Lancaster University, Lancaster, United Kingdom

**Keywords:** eye-tracking, memory, art gallery, museum, visibility, co-visibility

## Abstract

The spatial arrangement of artworks is recognized as one of the key elements of exhibition design. The underlying assumption is that the layout can strengthen the impact of individual exhibits, because the way visitors visually engage with artworks affects how they are cognitively processed. This paper explores the influence of the exhibits’ visual properties on the visitors’ attention and their memory of artworks. Attention was recorded with the use of mobile eye-tracking and memory was measured by an unanticipated recognition test immediately after the visit. The paper analyses both the total amount of attention spent on interacting with each artwork, as well as the strategy through which attention was allocated: through primarily longer (“diligent”) looks, versus primarily shorter (“distracted”) glimpses. Results of two experiments demonstrate that the visibility and co-visibility of artworks affected the amount of attention allocated to them, and the strategy of attention allocation. While the amount of attention contributed to improving the recognition memory of pictures, the strategy of attention allocation did not. These findings demonstrate the power of the exhibition’s visual properties to influence the experience of museum visitors but also highlight the visitors’ ability to employ alternative viewing strategies without diminishing the cognitive processing of artworks.

## Introduction

The unique role of physical art galleries prevails despite the ease of online access to the arts from the comfort of one’s own living room. Therefore, exhibiting art in a physical and curated setting must carry a unique value that is impossible to realize with aspatial and self-curated means of viewing art. Key functions of the gallery’s curated spatial layout lie in affecting the visitors’ cognitive engagement (Robinson, 1928; Bitgood et al., 1988; Peponis et al., 2004) and in supporting the interpretation of exhibits (Wineman and Peponis, 2010).

However, it is difficult to quantify the impact of the spatial layout on visitors’ cognitive processes. In controlled psychological experiments that study art viewing on a computer monitor, the challenge is to maintain ecological validity. In real (non-virtual) art exhibitions that are already designed to give more prominent locations to better artworks, the challenge is to isolate the influence of the physical setting on individual cognitive processes. The work here presented studies two processes (visual attention and memory), indicative of the visitor’s *engagement*. The key underlying argument is that it is the engagement (hereby defined as perceiving and cognitively processing artworks) that forms the necessary initial step for more complex understanding to arise. The paper addresses the challenge of disentangling the influence of visual properties of exhibitions from other factors affecting the visitor’s engagement with art exhibits.

To date, measuring the visitors’ engagement with artworks has been tackled by two research streams aiming to understand: (1) how visitors explore exhibits, and (2) how visitors understand and appreciate exhibits.

One line of research, known as “timing and tracking” studies (Diamond, 1999; Yalowitz and Bronnenkant, 2009; Westat, 2010, Dalton et al., 2012; for a historical review see Kirchberg and Tröndle, 2012), investigated whether the number of visible pictures affects the time visitors spend looking at them. Robinson (1928) conducted laboratory-based experiments where he varied the number of pictures simultaneously presented to participants. He found that the time spent on observing a single picture did not decrease proportionally to the number of presented artworks and that the most effective way to prolong the viewing time was to present the artwork in a complete isolation (Robinson, 1928). This was followed by museum-based observations of Melton (1935) who noticed that people stopped in front of a smaller number of paintings as the number of artworks in the gallery increased, but that their viewing time per painting remained close to 10 s. He suggested that artworks “compete” for the visitors’ attention (Melton, 1935). Bitgood et al. (2013) revisited these results arguing that the competition is driven not only by the content of pictures, but also by the subjectively perceived potential value of engaging them; and that this value fluctuates with the visitor’s distraction, fatigue, satiation, and selectivity. These factors can be influenced by the size and design of the exhibition space. For instance, larger exhibitions are walked through faster (Serrell, 1997). However, the viewer’s exploration strategy also plays an important role: visitors who traveled through fewer sections of the Louvre museum were shown to spend more time inside, than those who “rush through” a larger number of spaces (Yoshimura et al., 2012). It thus seems, that museum visitors have some limited amount of cumulative engagement time they are ready to “spend” on exploring the exhibits and that they adjust their exploration strategy *in situ* in order to accommodate this.

Trying to explain how such *in situ* adjustments take place, Smith and Smith (2001); see also: Smith et al., 2017) manually recorded the visitors’ stopping behavior in front of artworks. They observed a repeatable pattern consisting of two phases: the initial period of viewing shorter than 10 s (when possibly the decision is being made about stopping for longer or progressing forward) and the period of diligent viewing, averaging to about 30 s per picture, but greatly varying across them. However, such manual recordings [just like other measures of engagement in timing and tracking studies: reading interpretive text (Bitgood and Patterson, 1993), or having a conversation about the exhibit (Bitgood, 1993)] are likely to be biased toward detecting an already *diligent* engagement. They might ignore (or not explicitly distinguish) the role of shorter, haphazard interactions with the artworks, for instance occurring when visitors only glimpse at a painting without clearly stopping in front of it. In contrast, the aim of the current paper is to record *any* visual engagements with the exhibits (both diligent and not) and measure their impact on the recognition memory of artworks. We hypothesized that the visitors can vary their distribution of “haphazard” and “diligent” interactions independently of their cumulative viewing time and that the two variables are influenced by separate visual properties of the gallery space. Given this aim, we studied the visual attention directly, by recording the viewer’s eye movement.

This approach can be traced back to Buswell (1935) who recorded scan paths of his participants’ eye movement on an analog film while they were examining artworks inside his laboratory. After observing that the viewers’ attention was not uniformly distributed, he suggested: “it is probable that most of the visitors to an art gallery look at the pictures with this (quick survey) type of perception and that they see only the main centers of interest” (Buswell, 1935, p. 142). With the availability of mobile eye-tracking devices, today similar hypotheses can be investigated *in situ* (Hayhoe and Ballard, 2005; Tatler and Land, 2015; Kiefer et al., 2017).

Wessel et al. (2007) recorded the eye movement of three students during a small exhibition, showing that they employed a two-staged viewing strategy: first, they visually skimmed through a larger area (e.g., an entire wall), and only then analyzed individual exhibits in detail. Heidenreich and Turano (2011) asked four participants to view 14 paintings in the Baltimore Museum of Art, but found no significant correlations between eye fixations and the painting’s “saliency map” (i.e., a calculated prediction of the most visually salient areas of the image; Itti and Koch, 2000), nor between viewing times and the subsequent esthetic judgments of the artworks (see also Isham and Geng, 2013 for a similar result). It therefore seems that the two-stage viewing process suggested by Smith and Smith (2001) is reflected in the eye movement of gallery visitors, but that it cannot be explained by the visual saliency of artworks.

While “timing and tracking” studies investigated visitor behavior *in situ*, the field of experimental esthetics investigated how people form a deep impression of artworks, in controlled laboratory-based settings. It has broadly adopted the assumption about the two-stage nature of this process. For instance, the cognitive model of esthetic appreciation proposed by Leder et al. (2004) distinguishes between “automatic” and “deliberate” cognitive processes. According to this framework, when people see an artwork, its perceptual features and implicit memory relations are first analyzed by automatic cognitive processes. Only in the second phase, deliberate processes (such as domain specific expertise and contextual interpretation) become involved in the appreciation. Another model (Locher et al., 2007) also emphasized the two-stage nature of the esthetic experience, differentiating between a quick, automated decision (a “gist”), and longer, diligent viewing. In the supporting experiment, the participants’ eye movement was recorded alongside think aloud protocols while they were rating artworks for pleasingness. Viewers spent 32.5 s on average before doing so - a duration in line with Smith and Smith’s results (2001) and interpreted as the indication of the external validity of the model (Locher et al., 2007). Similarly, Bitgood (2010) presented a model adapted to the context of an entire art gallery visit. According to his “capture-focus-engage” framework, art appreciation is also sequential and the initial “capture” and “focus” stages do not involve deeper cognitive processing of the artwork. However, it is unclear where would lie the threshold between the initial “gist” phase and the deeper engagement. In Locher et al. (2007) studies, 2 s have passed before participants began to verbally describe the holistic features of an artwork. They also based their impressions on those image parts that they saw within the first 3 s of viewing. In line with this, Bitgood (2010) proposed that at least “a few seconds” must past before the viewer progresses beyond the “focus” phase to a deeper engagement.

In approaching and engaging art, the museum space surrounding it plays a key role (Newhouse, 2005; Hillier and Tzortzi, 2007; Zamani, 2009). The constellation of factors creating the difference between a museum and “any other” space has been jointly referred to as the “museum context.” To show its relevance, Brieber et al. (2014) asked two groups of participants to freely view the same art exhibition either in an art gallery or on a computer screen in a psychological laboratory. Participants inside the museum viewed pictures for longer. In a different study, Brieber et al. (2015) demonstrated that the same artworks were remembered better, liked more, and rated as more arousing, positive, and interesting when viewed in the real museum, compared to a computer-based simulation. Tröndle et al. (2014) recorded psychophysiological responses indicating arousal of visitors in a public art gallery. They observed that reactions were negligible when an artwork was hung just outside the entrance to the designated exhibition area and that they increased immiedietly after participants crossed the entrance. These studies demonstrate the effect of the “museum context.” It remains unclear, however, which aspects of the museum’s physical environment have the largest impact on the visitors’ experience.

One considered variable are the visual properties of the artworks’ hanging locations. The visibility and accessibility of exhibits were shown to predict the visitors’ stopping behavior and engagement (Bitgood et al., 1988; Hillier et al., 1996; Peponis et al., 2004). As demonstrated in the wayfinding literature, objects that are more visible in space are also more likely to be remembered (von Stülpnagel and Frankenstein, 2015). However, in the context of a museum visit, not all visibility is equally impactful. Stavroulaki and Peponis (2003) proposed that frontal visual catchment areas (quantified as 60° visibility cones extending from the front of the artworks) have particularly strong impact on the visitor experience.

Another spatial aspect attracting the researchers’ interest has been the co-visibility of exhibits. Bitgood et al. (1988) demonstrated that “competition” that arises from two exhibits being potentially co-visible by the viewer decreases the visitors’ stopping probability (see also: Melton, 1972 and Bitgood et al., 2013). However, co-visibility can also enrich the visitors’ experience. Lu and Peponis (2014) systematically modified the co-visibility of artworks in a virtual reality simulation of an art gallery. In their experiment, participants freely explored the virtual exhibition and graded its “clarity.” The co-visibility of artworks was correlated with a better understanding of the exhibition. The above studies therefore suggest that co-visibility might have a negative impact on the amount of attention allocated to individual exhibits, but that it can also simultaneously enhance the visitor’s deeper cognitive processing of the exhibition’s content.

The aim of the current work is to quantify the influence of the visibility and co-visibility of exhibits on the visitors’ cognitive engagement. This is achieved by: (a) dissociating the influence of the spatial location from the influence of the individual artwork, (b) systematically varying the visibility and co-visibility of artworks, and (c) jointly measuring the strategy of attention allocation, the cumulative amount of the allocated attention, and the memory resulting from it.

This manuscript expands on two earlier publications, which separately reported that eye-movement is correlated with the visibility of artworks’ locations (Krukar and Conroy Dalton, 2013) and that the overall spatial layout of a gallery affects the memorability of artworks (Krukar, 2014). It includes previously unreported data from Krukar (2014) and an entirely new experiment. The paper is therefore the first to ever consider the causal relation between the museum space, the visitor’s visual attention, and the resulting memory of artworks, in a single statistical model.

We hypothesized that the visual properties of exhibits (their visibility and co-visibility) affect both (a) the amount of attention (total viewing time per picture) and (b) the strategy with which attention is allocated to exhibits (haphazard vs. diligent viewing). We further expected that pictures that are viewed for longer and in a less haphazard manner, will be memorized better. It is thus proposed that the influence of visual properties of exhibits on the cognitive processing of artworks is mediated by the allocation of visual attention.

## Materials and Methods

Both experiments reported below employed the same procedure. Participants (recruited from the general population through public advertisement; but not artists, curators, nor architects) individually explored a non-public, mock-up art gallery. They were asked to wear a mobile eye-tracker during their visit and given the instruction to explore the space “as you would explore a regular art exhibition” for maximum 30 min (although they were not interrupted until 35 min – a limit imposed by the battery capacity of the eye-tracker). Participants were specifically asked to enter each space and to look at each picture. After exiting the gallery, they performed an unanticipated recognition memory test on a computer. Both studies received university’s ethical clearance. All participants signed an informed consent form and were paid 6 GBP.

**FIGURE 1:**
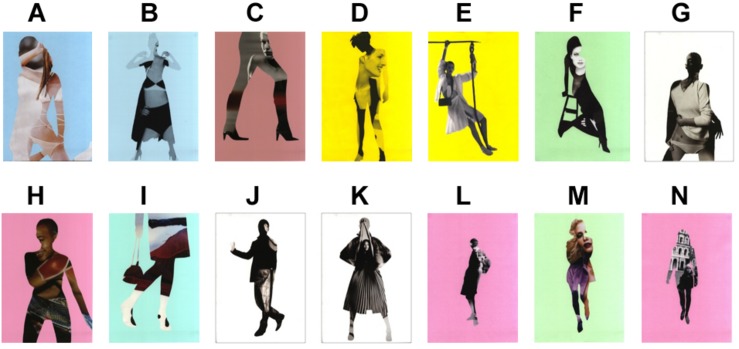
Artworks used in the experiments. “Strike a Pose”: www.susibellamy.co.uk. Used with permission. Labels **(A–N)** were added by the researcher for the purpose of distinguishing artworks in the data analysis (labels were not shown to participants).

Artworks displayed in the gallery were digital collages of equal dimensions (portrait-oriented A3), created by the artist Susi Bellamy (Figure 1). No labels or textual information about the artworks were provided and no other distractors were present in the gallery. As all artworks were untitled, any potential labels would be identical in content – their role in affecting the visitors’ engagement would therefore, most likely, be similar for each exhibit. The artworks were hung on locations which were kept identical within each experimental condition. However, the order in which each artwork appeared in the gallery (i.e., the placement of individual artworks at any given location) was randomized for each participant. Two participants exploring the gallery within the same experimental condition saw the same locations on the walls being occupied by artworks, saw the same set of artworks, but the exact artwork hanging on each location differed. This experimental design made it possible to distinguish the influence of spatial location and the influence of the individual picture. The subsequent data analysis involved three aspects: the analysis of the visual properties of artwork locations, the analysis of the eye movement, and the analysis of the recognition memory of artworks.

### Visual Properties

Two variables were used to analyze the visual properties of each artwork location:

#### Visibility Catchment Area (VCA)

The area size of a 60° visibility cone extending from the center of the artwork. This angle was selected based on previous work of Stavroulaki and Peponis (2003), as it permits distinct and undistorted viewing. The analysis was conducted using the DepthMapX software (Varoudis, 2012) and the area was measured in the arbitrary software units. The resulting numbers were transformed into z-scores so that their mean equaled 0, and the unit was their standard deviation. This makes the variable relative to the size of other VCAs in the studied gallery.

#### Co-visibility

The number of other artworks visible from the given location. The number is calculated from the central point of the artwork on the wall. Note that this measure only approximates the number of potentially co-visible artworks, since the visitor would need to stand with their back to the wall in order to see all other co-visible artworks. This method is chosen because it is independent of the path taken by participants and of their standing position.

### Eye Movement

Eye movement was recorded with the Tobii Glasses 1 mobile eye-tracker, sampling eye movement at 30 Hz. Fixations were detected using the built-in algorithm. Three variables were used to analyze eye movement:

#### Dwell Time (Amount of Attention)

Total cumulative time (in seconds) that the person spent looking at each artwork. In the reported experiments, it ranged from 1 to 303 s per picture, and the mean values per picture in all reported conditions were close to 30 s. Total dwell time has been previously linked to the meaning of the viewed area, its informativeness and interest (Holmqvist et al., 2011), as well as to the memory of the object’s position (Tatler et al., 2005).

#### Normalized Dwell Time (Relative Amount of Attention)

The percentage of total dwell time allocated by each participant to each individual artwork, in relation to the total time spent by them inside the gallery. The measure ranges from 0 to 100%. A participant spending an equal amount of attention on each artwork (and not dwelling on any non-artwork parts of the building) in Experiment 1, would achieve the value of (1/14) × 100 = 7.14% for each artwork. This measure indicates the relative amount of attention allocated to each exhibit while preserving the distinction between viewers who spent a large proportion of their viewing time fixating on exhibits and those who fixated on other elements of the environment (e.g., for the purpose of navigation or due to distraction).

Normalized dwell time was used in the analysis of Experiment 1 where the interest lies in the relative amount of attention in comparison to other exhibits. Raw dwell time was used in Experiment 2 where the differences between individual exhibits were not in the center of interest.

#### Dwell Ratio (Strategy of Attention Allocation)

The relation between the number of dwells longer than 2 s to dwells shorter than 2 s, per participant, per artwork. A single dwell is the time from the moment the participant’s scan path entered the boundaries of the image until it left them. A typical participant therefore employed more than one dwell on each artwork. For example, if there were 8 dwells in total, and five of them lasted longer than 2 s, while three of them lasted less than 2 s, the dwell ratio would equal 5/8 = 0.625. This metric ranged between 0 and 1. A higher number indicated that most visual engagements were longer (“diligent”). A lower number indicated that most interactions with the artwork occurred through short, haphazard glimpses.

This measure is based on the qualitative difference suggested to exist between different modes of art viewing (Smith and Smith, 2001; Leder et al., 2004; Locher et al., 2007; Bitgood, 2010) and describes the strategy with which attention was allocated to each exhibit. However, it is not implied that any particular strategy is undertaken intentionally. Although other ways to classify dwell types are possible, the current method has been selected in order to integrate the insight from observational visitor studies and laboratory-based experimental esthetics. Note that employing a different classification could affect the reported results.

Eye-tracking videos were coded manually and the interrater reliability analysis was carried out on 10% of the video material. Cohen’s Kappa was 0.99 for the total dwell time variable, and 0.98 for the normalized dwell time.

### Recognition Memory

Recognition memory was assessed using a computer-based image-discrimination task administered using the OpenSesame software (Mathôt et al., 2012). Participants were presented with pictures appearing sequentially, in a random order. Their goal was to answer whether they saw the picture inside the gallery by pressing the key marked as “yes” or “no.” The image set included all artworks present in the gallery, as well as the matching number of foils (new pictures, not present in the gallery). Foils were created from other artworks of the same artist, and by manipulating the graphic properties of the images present inside the gallery.

In both experiments, the ratio between response times and accuracy was stable across conditions, confirming no uneven speed-accuracy trade-off (Kahana and Loftus, 1999). This makes it possible to analyze the accuracy of responses as a key variable of interest. Using this measure in a linear mixed-effect model does not require aggregating the data by-participants (e.g., unlike the d’ metric), making it possible to simultaneously consider the differences in accuracy between participants, between stimuli, and between the original locations of their corresponding artworks in the gallery.

## Experiment 1

### Experimental Design, Procedure, and Participants

**FIGURE 2:**
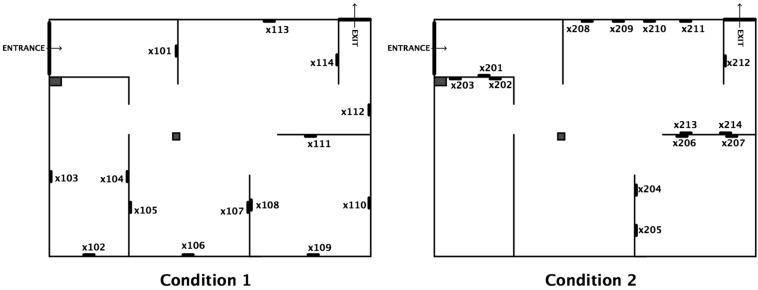
Two conditions arranged within Experiment 1, together with unique identifiers of artwork locations.

**FIGURE 3:**
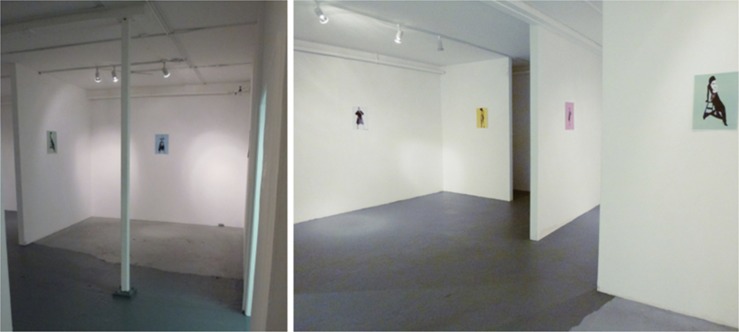
Views from the mock-up art gallery used in Experiment 1.

**FIGURE 4:**
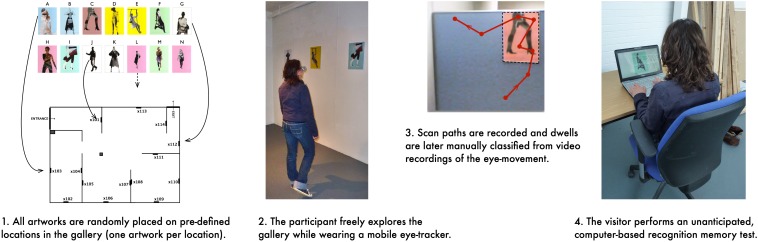
An overview of the experimental procedure.

Two experimental conditions were arranged (Figures 2, 3). The shape of the gallery walls was identical in both conditions but the spatial arrangement of artwork locations differed. Table 1 summarizes their VCA and co-visibility measures. Condition 1 included only one artwork per wall, while Condition 2 consisted of a denser arrangement of artworks on individual walls, with more walls being empty. Participants were randomly allocated to one of the two conditions and invited to explore it while wearing Tobii Glasses 1. After disregarding recordings with poor eye-tracker calibration (4 participants), the data of 28 participants were included in the analyses (Cond. 1: 13, Cond. 2: 15; of which 3 and 8 females, respectively; participants’ age range: 21–47 and 20–63, respectively). Figure 4 presents an overview of the procedure.

**TABLE 1:** Visual properties of all locations in Experiment 1 (VCA is provided in arbitrary units of the DepthMapX software, which were standardized and mean-centered for the purpose of further analyses).

Condition 1			Condition 2		
	
Location	VCA	Co-visibility	Location	VCA	Co-visibility
x101	52121	1	x201	16829	0
x102	110788	2	x202	130217	1
x103	48408	3	x203	95532	1
x104	32137	2	x204	72764	5
x105	112856	6	x205	59763	5
x106	235743	3	x206	71270	3
x107	70784	2	x207	58939	3
x108	71857	4	x208	212837	5
x109	125122	3	x209	224943	5
x110	161844	3	x210	194981	7
x111	73819	4	x211	129182	7
x112	198118	0	x212	5259	1
x113	186388	2	x213	60790	5
x114	164517	5	x214	56346	6

### Data Analysis

The current paper focuses on the individual influence of each location on eye-movement and recognition memory. Both conditions are only analyzed here as a means of assessing whether the behavioral patterns hold in two different spaces. In that framework, systematic differences distinguishing the two conditions do not confound the linear effect of VCA and co-visibility on attention and memory.

The artwork’s location influences how much visual attention it attracts (and what kind of attention); this attention in turn influences how well the artwork is remembered. Visual attention variables (normalized dwell time and dwell ratio) are dependent variables influenced by visual properties of artwork locations; however, they can also be treated as independent variables (predictors) of the recognition memory performance. In order to statistically verify this causal relation, Piecewise Structural Equation Modeling (SEM) is used (Lefcheck, 2016). The main difference between Piecewise SEM and traditional SEM is the consideration of random effects within the framework of linear-mixed effect models. Three random effects are considered in the main analysis: the by-participant random effect (individual participants’ responses are likely to be related), the by-location random effect (responses to objects hanging on the same location are likely to be related), and the by-picture random effect (responses to the same picture are likely to be related). This approach does not require aggregating the data across the participants or across the locations.

The constructed Piecewise SEM evaluates whether the visual properties of individual locations influence the amount of attention each participant allocated to the artwork currently hanging on that location and, in turn, how likely the person was to recognize this artwork in the subsequent memory test. The tested structure initially consisted of three models, presented in Table 2 (two linear mixed-effect models, and one mixed-effect model with a binomial link for explaining the categorical variable of reaction time accuracy; random effects structure is denoted in the form accepted by the lme4 R package; Bates et al., 2015).

**TABLE 2:** Mixed-effect models included in the SEM analysis of Experiment 1.

Model	Response	Fixed effect predictors	Random effects structure
m1	Norm. dwell time	VCA + co-visibility	(1| location) + (1| participant)
m2	Dwell ratio	VCA + co-visibility	(1| location) + (1| participant)
m3	Memory accuracy	norm. dwell time + dwell ratio	(1| location) + (1| participant)

The influence of individual pictures was initially considered but disregarded: each model was tested against alternative structures of random effects. Considering the random by-picture effect, either instead of, or together with the by-location effect, did not improve the models’ fit. The random effect structure that consistently provided the best fit was the one including the random by-participant effect and the random by-location effect. Moreover, including the effect of condition in the random effects structure did not improve the fit of any of the models, suggesting that the relation between the strategies of attention, amount of attention, and memory is similar in both conditions.

### Results

#### Descriptive Statistics

Participants spent between 1 and 29 min inside the gallery. The mean time spent inside was 10:15 min in Condition 1 and 7:54 min in Condition 2 (difference not statistically significant when measured by the Wilcoxon rank sum test: *W* = 139, *p* = 0.444). Individual visitors dedicated between 52 and 94% of their time inside to looking at artworks (as opposed to looking at other elements of the environment, e.g., for the purpose of navigation or due to distraction). This difference between conditions was not statistically significant (Cond. 1: *M* = 81%, Cond. 2: *M* = 72%, *W* = 146, *p* = 0.077). Mean normalized dwell time (per picture and per participant) was 5.47%. The dwell ratio ranged from 0.24 to 0.60 per participant, with the mean value of 0.37 in Condition 1 and 0.42 in Condition 2 (*W* = 72.5, *p* = 0.14). Recognition memory accuracy ranged from 0.36 to 1.00 when aggregated by-participant, from 0.64 to 1.00 when aggregated by-picture, and from 0.67 to 1.00 when aggregated by-location (further analyses account for these effects simultaneously). Considering all interactions, across all participants and both conditions, the average total viewing time of a single artwork was 32 s, which validates our procedure in-line with visitor observations in “timing and tracking” studies inside working art galleries (Smith and Smith, 2001; Smith et al., 2017). Average recognition memory accuracy was 0.78. Three participants did not look at all artworks, missing a single artwork each (picture D at location x201, G at x212, and F at x212); although it is possible that an existing glimpse was too short to be registered by the eye-tracker. As linear-mixed effect models include by-participant random effects, we decided not to remove outliers based on any subjectively chosen threshold (e.g., of minimum visit time), but instead let the statistical model to adjust for extreme outcomes through the statistical process of shrinkage.

#### Modeling the Interaction of the Visual Properties, Attention, and Memory

Statistical models that were used in the Piecewise SEM are separately summarized in Table 3. They demonstrate that the more visible an artwork was (i.e., the larger its VCA), the more dwell time it attracted, relative to other artworks: increasing VCA from 0 to 1 *SD* resulted in attracting 0.51% more normalized dwell time. Considering that the difference between VCAs of individual locations ranged from −1.57 *SD* to 1.97 *SD*, it can be estimated that the difference between the least and the most visible location in normalized dwell time caused by the effect of visibility alone (while controlling for other by-location effects) is 1.80%. Higher VCA was also associated with lower dwell ratio: an increase in VCA from 0 to 1 *SD* caused lowering the dwell ratio by 0.09, meaning that participants looked at more visible locations in a more “distracted” manner.

**TABLE 3:** Piecewise SEM relating spatial predictors, visual attention, and recognition memory in Experiment 1 (R^2^ estimates the proportion of variance in the data explained by the fixed factors alone; Conditional R^2^ estimates the proportion of variance explained by the fixed and random factors).

Model	R^2^	Cond. R^2^	Response	Predictor	Estimate	Std. error	*p*-value
m1	0.03	0.09	Norm. dwell time	VCA	0.508	0.183	**0.010**
			Norm. dwell time	Co-visibility	−0.017	0.091	0.853
m2	0.18	0.36	Dwell ratio	VCA	−0.090	0.018	**<0.001**
			Dwell ratio	Co-visibility	−0.022	0.009	**0.025**
m3	0.07	0.18	Memory accuracy	Norm. dwell time	0.250	0.077	**0.001**
			Memory accuracy	Dwell ratio	0.763	0.573	0.183
Corr. err.			Norm. dwell time	Dwell ratio	0.143	–	**0.002**

*Significant *p*-values lower than 0.05 are marked in bold.*

Also the co-visibility had a significant effect on decreasing the dwell ratio: each additional 1 co-visible artwork was associated with a decrease in dwell ratio by 0.02. Since co-visibility in the studied locations varied from 0 to 7, this translated to a difference of 0.16 between dwell ratios of the least and the most co-visible artwork in the galleries. This effect can be related to the influence of the co-visibility alone, while controlling for VCA and for other unexplained (random-effect) differences between locations. More co-visible artworks were engaged with in a more distracted manner - through a lower proportion of diligent dwells.

Recognition memory accuracy was significantly predicted by normalized dwell time, but not by the dwell ratio. Each additional 1% in the normalized dwell time was associated with a change in the odds ratio of correctly remembering the picture, equal to 1.28:1. Odds ratios are calculated by taking the exponent of the estimated coefficient and can be interpreted similarly to betting odds; it can be thus said that for a 1 unit increase in the normalized dwell time, we expect a 28% increase in the odds of remembering an artwork.

**FIGURE 5:**
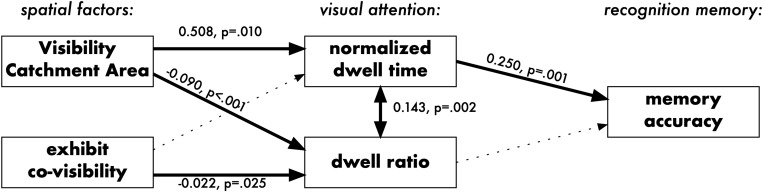
Piecewise SEM relating spatial predictors, visual attention, and recognition memory in Experiment 1. Dotted lines represent non-significant pathways. Numbers are the estimated effects of the linear model: e.g., an increase by 1 SD in Visibility Catchment Area (VCA) is associated with an increase by 0.508 in normalized dwell time.

The final Piecewise SEM is presented in Figure 5. A d-separation test for missing paths (Shipley, 2013; Lefcheck, 2016) indicated that the model should also include the correlation of the normalized dwell time with dwell ratio, meaning that the longer each participant looked at a particular picture, the higher proportion of those dwells were “diligent”. As this correlation does not have a clear causal direction, it has been modeled in the form of a correlated error (Lefcheck, 2016). The model’s goodness-of-fit was verified by the Fisher’s C statistic being non-significant (C = 2.00, *p* = 0.735), meaning that the model represents the data well. It can be summarized that visual properties of the exhibits’ locations affected the memory accuracy of artworks indirectly: by influencing the visual attention.

#### Differences Between Locations

**FIGURE 6:**
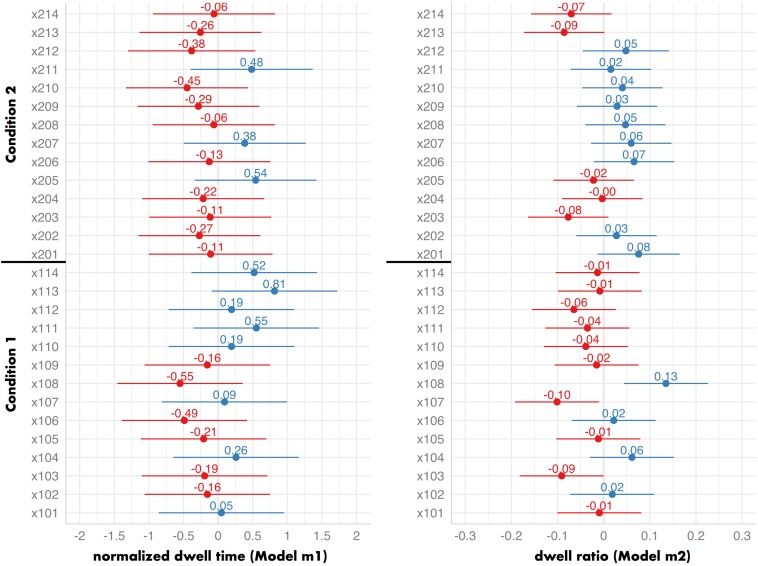
A forest plot of random by-location effects in Experiment 1 (left: Model m1; right: Model m2), with 95% Confidence Intervals. It demonstrates how the models’ predictions are affected by each individual location. If location had no effect on visual attention, these values would equal 0.

The main effect of VCA and co-visibility can be interpreted despite other possible differences across the locations (e.g., due to other spatial variables not considered in the model) and across the conditions. Nevertheless, it is valuable to investigate the differences between individual locations in detail, as they might reveal systematic biases in how visitors engaged with them. Figure 6 presents a forest plot of the random effect of locations (Lüdecke, 2017) in Models m1 and m2 visualizing the influence that each location has on the dependent variable after considering the effects of VCA and co-visibility. For example, the value of 0.48 for the location x211 in the Model m1 (dependent variable: normalized dwell time) indicates that objects hanging at this location are predicted to receive the normalized dwell time higher by 0.48 after considering the fixed effects of VCA and co-visibility. If location had no effect on visual attention, these values would equal 0. Location x113 attracted the highest amount of attention (i.e., on average it was viewed the longest, compared to other locations); location x108 attracted the least attention (i.e., on average it was viewed the shortest, compared to other locations).

## Discussion

Results demonstrate that accounting for only two variables (visibility and co-visibility of artworks) can explain a significant portion of the variance in the visitors’ behavior. Data show that visual properties of artworks’ locations had a significant influence on the relative amount of attention allocated to artworks (normalized dwell time in Model m1) and on the strategy of attention allocation (dwell ratio in Model m2). The amount of attention had a significant influence on the memory accuracy, but the strategy of attention allocation did not (Model m3). This demonstrates that local visual properties of hanging locations in an art gallery directly affect the amount of attention each artwork receives, the strategy of attention allocation and, in turn, indirectly affect the memory of the engaged artworks.

Results confirm that more visible locations attract more attention - a finding made earlier in the context of zoos and science exhibits (Bitgood et al., 1988; Peponis et al., 2004). However, after controlling for other confounding factors, this effect was small: the difference between the least and the most visible locations that can be associated with the isolated effect of VCA was 1.80% in the normalized dwell time. Further, Model m2 had a considerably higher *R*^2^ value, compared to Model m1. This shows that the strategy of attention allocation was affected more, compared to the amount of attention: Modifying visibility and co-visibility of artwork locations in a gallery bears a limited change to how long visitors will engage with them but a more consistent change to their viewing strategy. It is thus likely that the amount of attention is driven primarily by the individual preferences of each viewer (Brieber et al., 2014) - an assumption that is not tested in the current paper as we explicitly focused on cognitive engagement and not subjective preferences in art viewing.

The influence of space on recognition memory is indirect, and it seems to be driven primarily by the mediating role of the amount of allocated attention. Artworks hanging at more visible locations are remembered better *because* they are viewed for longer. This causal path is an important property of the human-environment interaction in the art gallery because it can be very sensitive to interference. Many elements of the visit (e.g., visitors’ goals, additional interpretive material), can disrupt the carefully planned spatial influence envisioned by the curator.

While the recognition memory performance was directly influenced by the proportion of attention allocated to individual artworks, it was not significantly predicted by the dominant strategy of attention allocation. This result is counterintuitive and contrary to the dominant assumption in the visitor studies literature, which traditionally tends to link this strategy of attention allocation with the inferior understanding of the exhibition. What could be interpreted as a more “distracted” viewing behavior did not jeopardize the cognitive processing of the artworks’ content.

The exact benefit of increasing the relative visibility of artworks is difficult to judge because there seem to be two contrary processes at play at the perceptual stage of the interaction. More visible locations were likely to be looked at in a more distracted way but, simultaneously, participants looked at more visible locations for proportionally longer. Experiment 2 was designed to disentangle the effect of the two studied visual properties (VCA and co-visibility).

## Experiment 2

### Experimental Design, Procedure, and Participants

**FIGURE 7:**
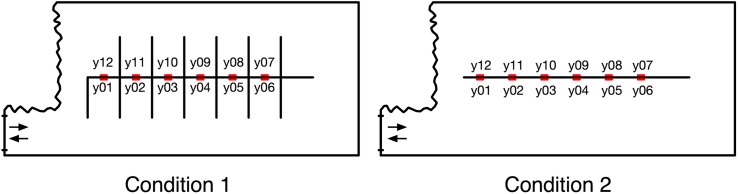
Two conditions arranged within Experiment 2.

**FIGURE 8:**
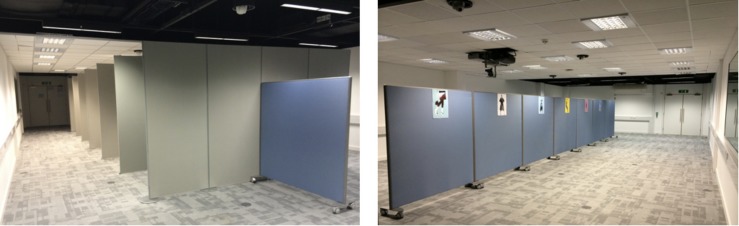
A view from Condition 1 **(left)** and Condition 2 **(right)** arranged within Experiment 2.

Two layouts were created in a different space than the one used in Experiment 1. In both conditions, VCAs of all locations were identical (Figures 7, 8). The co-visibility of each artwork varied across the conditions (co-visibility = 0 in Condition 1 and co-visibility = 5 in Condition 2). Twelve, instead of 14 pictures from the same set were used in this study (excluding pictures G and H from Figure 1). Compared to Experiment 1, the artworks’ number was reduced, in order to preserve an empty wall section at the far end of the artwork sequence - this was intended to prevent participants from rapidly turning around the wall and spotting the first image on the opposite side of the wall from an unusually close distance which could confound the uniformity of otherwise similar locations. The procedure followed that of Experiment 1. Participants were randomly allocated to one of the two conditions and the location of artworks was randomized for each visitor. After disregarding uncomplete recordings (of 16 participants in total, due to faulty battery slot of the eye-tracker and poor eye-tracker calibration due to technical issues with lighting), the data of 29 participants were included in the analyses (Cond. 1: 10, Cond. 2: 19; of which 6 and 14 females, respectively; participants’ age range: 26–68 and 23–67, respectively). Most statistics are calculated on *participant-artwork interactions*, i.e., on (10+19) participants x 12 artworks = 348 data points.

### Data Analysis

Unlike in Experiment 1, the main source of variation in the influence of visual properties lay across the conditions, not across the locations. For this reason, the data analysis based on Piecewise SEM was altered compared to the procedure described for Experiment 1. Firstly, the visual predictors (VCA and co-visibility) were substituted by the categorical variable distinguishing between Conditions 1 and 2. Secondly, normalized dwell time was substituted by the raw dwell time, as it is more informative to compare two conditions on a non-relative scale (i.e., total dwell time in seconds). However, the raw dwell time measure is confounded by the time spent inside the gallery (which differed substantially across the individuals and across the conditions). It can be expected that pictures are viewed for longer by those participants, who spend more time inside the gallery. In order to account for this, time inside the gallery (per participant) was included as a control variable in the models presented below. This way, all other effects (for instance, the effect of experimental condition) can be evaluated even under the presence of noticeable differences in the times spent inside the gallery. Thirdly, the random effect of locations was substituted by the random effect of pictures, as little variation was expected to arise across so uniformly arranged locations.

The tested Piecewise SEM consisted of three models, presented in Table 4. Each model was tested against alternative structures of random effects. The random effect structure which consistently provided the best fit was the one including the random by-participant effect and the random by-picture effect. Considering the random by-location effect, either instead of, or together with the by-picture effect, did not improve the models’ fit.

**TABLE 4:** Mixed-effect models included in the SEM analysis of Experiment 2.

Model	Response	Fixed effect predictors	Random effects structure
m4	Dwell time	Condition (1 vs. 2) + time inside	(1| picture) + (1| participant)
m5	Dwell ratio	Condition (1 vs. 2) + time inside	(1| picture) + (1| participant)
m6	Memory accuracy	Dwell time + dwell ratio + time inside	(1| picture) + (1| participant)

### Results

#### Descriptive Statistics

Participants spent between 2 and 35 min inside the gallery. Mean time spent inside was 8:25 min in Condition 1 and 7:39 min in Condition 2 (*W* = 112, *p* = 0.456). Visitors dedicated between 56% and 97% of their time inside to looking at artworks (and the difference between conditions was statistically significant; Cond. 1: *M* = 78%, Cond. 2: *M* = 87%, *W* = 39, *p* = 0.009). The dwell ratio ranged from 0.13 to 0.95 per participant, with the mean value of 0.71 in Condition 1 and 0.40 in Condition 2 (a significant difference, as demonstrated in statistical models below). Recognition memory accuracy ranged from 0.50 to 1.00 when aggregated by-participant, from 0.76 to 0.98 when aggregated by-picture, and from 0.75 to 0.94 when aggregated by-location. When to consider all interactions, across all participants, and both conditions, the average total viewing time of a single artwork was 34 s, which is in-line with *in situ* observations of museum visitors (Smith and Smith, 2001; Smith et al., 2017). Average recognition memory accuracy was 0.86. All participants looked at each artwork at least once.

#### Modeling the Interaction of the Visual Properties, Attention, and Memory

Models explaining the relation between the experimental conditions, visual attention measures, and memory are summarized in Table 5. Results demonstrate that the dwell time was associated with the experimental condition: participants in Condition 2 viewed each artwork for 3.8 s longer on average, after controlling for the effect of the total time spent inside the gallery. Unsurprisingly, time spent inside the gallery was also a significant predictor of dwell time per picture: each additional minute of the gallery time contributed to 4.7 s of viewing, on average, per artwork. Dwell ratio was lower in Condition 2 by 0.30, meaning that participants in Condition 2 employed a viewing strategy based on a lower proportion of long dwells - their viewing behavior was more “distracted”. Recognition memory was predicted by dwell time but not by the dwell ratio. Each additional second of dwell time was associated with the increase in odds of remembering an artwork by 6%; 10 s of additional dwell time would increase the odds of correctly remembering the artwork by 76% (i.e., to 1.76:1). Time spent inside the gallery had a negative relation with the recognition memory (but not statistically significant): each minute in the gallery was associated with a decrease in the odds of remembering an artwork by 15% (0.85:1).

**TABLE 5:** Piecewise SEM relating two spatial conditions, visual attention, and recognition memory in Experiment 2.

Model	R^2^	Cond. R^2^	Response	Predictor	Estimate	Std. error	*p*-value
m4	0.78	0.79	Dwell time (sec.)	Condition	3.796	1.348	**0.005**
			Dwell time (sec.)	Time inside (min.)	4.738	0.133	**<0.001**
m5	0.24	0.53	Dwell ratio	Condition	−0.304	0.066	**<0.001**
			Dwell ratio	Time inside (min.)	<0.001	0.007	0.980
m6	0.09	0.15	Memory accuracy	Dwell time (sec.)	0.057	0.020	**0.004**
			Memory accuracy	Dwell ratio	0.869	0.657	0.186
			Memory accuracy	Time inside (min.)	−0.168	0.087	0.054
Corr. err.			Dwell time (sec.)	Dwell ratio	0.123	–	**0.011**

*Estimates for conditions represent a predicted change from condition “1” to condition “2.” Significant *p*-values lower than 0.05 are marked in bold.*

**FIGURE 9:**
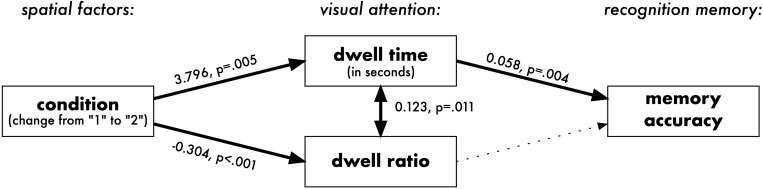
Piecewise SEM relating two spatial conditions, visual attention, and recognition memory in Experiment 2. Dotted lines represent non-significant pathways. All paths control for the total time spent inside the gallery by each participant (not visualized).

The final Piecewise SEM is depicted in Figure 9. A d-separation test indicated that the model should include the correlation of dwell time with dwell ratio, which was modeled in the form of a correlated error. The model’s goodness-of-fit was verified by the Fisher’s C statistic being non-significant (C = 0.46, *p* = 0.794), meaning that the model represents the data well. Considering the differences between individual locations (a random by-location effect) did not significantly improve the models’ fit and therefore is not reported in detail.

### Discussion

The Piecewise SEM demonstrates that the experimental condition significantly affected the strategy of attention allocation and the amount of attention dedicated to individual pictures. Participants in Condition 2 explored the pictures in a manner based on shorter glimpses but cumulatively looked at them for 3.8 s longer, on average. Participants in Condition 1 explored the artworks mainly through longer engagements. In line with the results of Experiment 1, the amount of attention spent on each picture had an impact on its recognition memory, but there was no corresponding evidence for the impact of the strategy of attention allocation (i.e., dwell ratio did not predict recognition memory accuracy).

Experiment 2 confirms and expands findings of Experiment 1 when explaining the relationship between the visual properties of space, visual attention, and memory. Co-visibility affected the visual attention of museum visitors. After considering the role of visual attention, no further effect of experimental condition on recognition memory was detected by the missing-path analysis. This suggests, that the key difference between the two spaces is in how they mediate attention. Similarly to the Experiment 1, however, the strategy of attention allocation had a negligible effect on memory, and it was primarily the dwell time that affected how well participants remembered individual artworks. This casts doubt on the common assumption of many cognitive models of the esthetic experience that make a qualitative distinction between the gist and the deeper cognitive processing of artworks. It is unlikely that appreciating art is based on a strictly sequential cognitive process (Nadal and Skov, 2017) and the presented experiments confirm that the role of “gist” in the processing of artworks should not be undervalued. Orienting in space (e.g., for navigational purposes) and haphazard glimpses are not a “distraction in”, but an intrinsic part of the cognitive experience of art gallery visitors. Researchers interested in studying the visitor experience in an ecologically valid manner should employ methods sensitive to the impact of short and seemingly haphazard interactions with the art.

The experimental condition also had an effect on viewing times per artworks, even after accounting for individual differences in the time spent inside the gallery - participants in Condition 2 (which caused “more distracted” viewing), cumulatively looked at pictures for longer. This is contrary to the classic findings of Robinson (1928) who suggested that a complete isolation of artworks increases their viewing times. In the current study, a complete isolation increased the proportion of long, “diligent” dwells but decreased the cumulative engagement times. One reason for this disparity might be the difference in methods used. Eye-tracking is sensitive to short, haphazard glimpses in the situated context of an art gallery visit. It was unavailable to Robinson and a similar effect is difficult to simulate in laboratory-based paradigms. Classical methods based on third-person observations of gallery visitors are likely to underestimate total dwell times accumulating from shorter interactions.

The findings also demonstrate the situated character of the visitors’ cognition inside distinct art gallery layouts. What could be seen as less optimal, or a “distracted” way of interacting with artworks, in fact resulted in enhancing the interactions. It is unclear, whether the visitors purposefully compensated for the “distracted” way of looking at images by engaging them for longer, or whether the prolonged viewing times are a “side-effect” of the more haphazard viewing behavior. The former could occur if short glimpses are not subjectively perceived as satisfactory, desired, or pleasant interactions (even though they implicitly contribute to strengthening the memory trace of artworks). The latter explanation is possible if an initial period of “gist” is a necessary element of any diligent engagement: some interactions in Condition 2 would therefore consist of the “gist” *alone*, while others would consist of the “gist” *followed by* a period of more involved viewing. An increased number of engagements would thus automatically be associated with a higher number of “gist”-periods, additively increasing the cumulative viewing times.

An important contribution of the presented data is expanding the traditional notion of the exhibits’ “competition” (Melton, 1972; Bitgood et al., 2013, 1988). In Experiment 1, location had a significant impact on visual attention (c.f., Figure 6). In Experiment 2, however, it did not (including the random by-location effect did not improve final models). This demonstrates that in Experiment 2 differences in the amount of attention allocated to individual pictures lay primarily between participants (e.g., their interests), and between pictures (e.g., their content). Such a contrast to the results of Experiment 1 shows that the competition can arise either between the spatial prominence of artworks’ locations (Experiment 1) *or* between the artworks’ content (Experiment 2). When the visibility and the co-visibility of individual artworks are diversified, the competition is driven by the spatial prominence (Experiment 1). When the visibility and the co-visibility of each artwork in the gallery are similar, the competition is driven by the content (Experiment 2).

## Limitations

While aiming to isolate the influence of spatial factors, this research studied pre-arranged environments, using a single set of artworks. This implies limitations that should be considered before applying the findings to the context of art galleries typically found outside research laboratories.

First, no interpretive text was included in the exhibitions. Visitor studies have repeatedly reported the crucial role that the presence and design of the interpretive text can have on the visitors’ engagement with the exhibits (Bitgood and Patterson, 1993; Bitgood, 1996, 2014). In the reported studies, as all artworks were untitled, all potential labels would be identical: their influence on the visitor experience would therefore likely be smaller, compared to exhibitions with diverse labels.

Second, the studied variables involved visual engagement with and the recognition memory of artworks. These should not be confused with a more diligent form of engagement (involving interpretation, reading, or talking about the exhibits) that have typically been the main focus of research in the visitor literature. While low-level cognitive processes such as those described in the current paper can be seen as the “building blocks” of the more holistic cognitive experience, they are not synonymous with it.

Third, only the works of a single artist were presented in the described studies. More work is necessary to verify whether similar patterns hold for other types of art (e.g., for more diverse types of images, sculptures, or media installations) and for exhibitions containing works of multiple artists.

Fourth, all reported analyses are path-independent. It is not claimed that individual paths, viewing sequences, or angles of approach do not have an impact on the visitor experience. From the curatorial point of view, however, it might be beneficial to understand the effect that the relative visibility and co-visibility of artworks have on the visitors when “all other things are kept equal.”

Fifth, the paper focuses on visual properties of artwork locations, and not on the shape of the gallery, its hallway topology, or the floor area size. The underlying reason is that the distribution of hanging locations is a factor most amenable to curatorial interventions.

Lastly, it bears noting that while a higher proportion of short glimpses has been discussed in the context of “distracted” engagement, it is possible that some of this behavior was driven by the explicit willingness to *compare* co-visible artworks. Distinguishing seemingly similar eye-movement data driven by these two distinct reasons lay beyond the scope of the current paper. While the current paper analyzed exhibitions as a set of separate artworks, it is their *synergy*, resulting in exhibitions becoming more than the “sum of their parts,” that remains a truly challenging question open to future work.

## Conclusion

The more visible an artwork was, the more attention it attracted. Artworks that were more co-visible, were viewed in a more haphazard way. However, more haphazard viewing strategy simultaneously resulted in higher cumulative viewing times and did not negatively affect the cognitive processing of artworks. Memory of artworks seems to be affected by the cumulative amount of attention allocated to them (including even short glimpses) but not by the strategy of attention allocation. The role of space in steering the visitors’ cognitive engagement is not so much to affect the amount of engagement, but rather to facilitate the strategy with which it occurs.

The strategy with which people view artworks is not necessarily going to affect the depth of their cognitive processing but can have other influence on the experience. Exploring an art exhibition is an embodied experience (Pallasmaa, 2013; Zisch et al., 2013), and what happens to our body–including the type of viewing behavior it was prompted to exert–is likely to be integrated into the retrospective evaluation of the visit. Space bears a profound influence on this aspect, by guiding the strategy with which attention is allocated. This happens both on the local level of individual exhibits (by creating differences between the exhibits’ visibility and co-visibility in space: Experiment 1), and on the global level of the entire exhibitions (in cases where the differentiation between individual exhibits is minimalized: Experiment 2).

Future research on the influence of museum space can benefit from mobile eye-tracking, as this method enables detecting subtle patterns of attention. Structural equation modeling and hierarchical modeling are appropriate techniques for studying the mediating character of attention (Hine et al., 2016). These methods demonstrated that what traditionally could be interpreted as a poor, “distracted” visitor experience, had little negative impact on the cognitive processing of artworks. Visitors were able to adjust their viewing strategies inside a potentially less optimal space. This finding supports the planning of more diverse spatial interactions with the art.

## Data Availability Statement

The datasets generated for this study are available at: https://osf.io/d6mwe/.

## Ethics Statement

The studies involving human participants were reviewed and approved by the Northumbria University. The participants provided their written informed consent to participate in this study.

## Author Contributions

JK and RD contributed to the conception and design of the studies, manuscript revision, and read and approved the submitted version of the manuscript. JK collected the data, performed the statistical analysis, and wrote the first draft of the manuscript. RD supervised the work.

## Conflict of Interest

The authors declare that the research was conducted in the absence of any commercial or financial relationships that could be construed as a potential conflict of interest.

## References

[B1] Bates D. Mächler M. Bolker B. Walker S. (2015). Fitting linear mixed-effects models using lme4. *J. Stat. Softw.* 67 1–48. 10.18637/jss.v067.i01

[B2] Bitgood S. (1993). Social influences on the visitor museum experience. *Visit. Behav.* 8 4–5.

[B3] Bitgood S. (1996). The role of attention in designing effective interpretive labels. *J. Interpret. Res.* 5 31–45.

[B4] Bitgood S. (2010). *An Attention-Value Model of Museum Visitors.* Washington, D.C: Center for the Advancement of Informal Science Education.

[B5] Bitgood S. (2014). *The Importance of Attention and Value for Interpretation.* Paris: The International Heritage Interpretation E-Magazine.

[B6] Bitgood S. McKerchar T. L. Dukes S. (2013). Looking back at Melton: gallery density and visitor attention. *Visit. Stud.* 16 217–225. 10.1080/10645578.2013.827024

[B7] Bitgood S. Patterson D. Benefield A. (1988). Exhibit design and visitor behavior: empirical relationships. *Environ. Behav.* 20 474–491. 10.1177/0013916588204006

[B8] Bitgood S. C. Patterson D. D. (1993). The effects of gallery changes on visitor reading and object viewing time. *Environ. Behav.* 25 761–781. 10.1177/0013916593256006

[B9] Brieber D. Nadal M. Leder H. (2015). In the white cube: Museum context enhances the valuation and memory of art. *Acta Psychol.* 154 36–42. 10.1016/j.actpsy.2014.11.004 25481660

[B10] Brieber D. Nadal M. Leder H. Rosenberg R. (2014). Art in time and space: context modulates the relation between art experience and viewing time. *PLoS One* 9:e99019. 10.1371/journal.pone.0099019 24892829 PMC4043844

[B11] Buswell G. T. (1935). *How People Look at Pictures.* Chicago, IL: University of Chicago Press.

[B12] Dalton N. S. Conroy Dalton R. Hölscher C. Kuhnmünch G. (2012). “An iPad app for recording movement paths and associated spatial behaviors,” in *Spatial Cognition VIII*, eds Stachniss C. Schill K. Uttal D. (Berlin: Springer), 431–450. 10.1007/978-3-642-32732-2_28

[B13] Diamond J. (1999). *Practical Evaluation Guide: Tools for Museums and Other Informal Educational Settings.* Walnut Creek, CA: Rowman Altamira.

[B14] Hayhoe M. Ballard D. (2005). Eye movements in natural behavior. *Trends Cogn. Sci.* 9 188–194. 10.1016/j.tics.2005.02.009 15808501

[B15] Heidenreich S. M. Turano K. A. (2011). Where does one look when viewing artwork in a museum? *Empir. Stud. Arts* 29 51–72. 10.2190/EM.29.1.d

[B16] Hillier B. Major M. Desyllas J. Karimi K. Campos B. Stonor T. (1996). *Tate Gallery, Millbank: A Study of the Existing Layout and New Masterplan Proposal.* London: University College London.

[B17] Hillier B. Tzortzi K. (2007). “Space syntax: the language of museum space,” in *A Companion to Museum Studies*, ed. Macdonald S. (Hoboken, NJ: Blackwell Publishing Ltd), 282–301. 10.1002/9780470996836.ch17

[B18] Hine D. W. Corral-Verdugo V. Bhullar N. Frias-Armenta M. (2016). “Advanced statistics for environment-behavior research,” in *Research Methods for Environmental Psychology*, ed. Gifford R. (Chichester: John Wiley & Sons, Ltd), 369–388. 10.1002/9781119162124.ch19

[B19] Holmqvist K. Nyström M. Andersson R. Dewhurst R. Jarodzka H. Van de Weijer J. (2011). *Eye tracking: A comprehensive guide to methods and measures.* Oxford: Oxford University Press.

[B20] Isham E. A. Geng J. J. (2013). Looking time predicts choice but not aesthetic value. *PLoS One* 8:e71698. 10.1371/journal.pone.0071698 23977115 PMC3745463

[B21] Itti L. Koch C. (2000). A saliency-based search mechanism for overt and covert shifts of visual attention. *Vis. Res.* 40 1489–1506. 10.1016/S0042-6989(99)00163-7 10788654

[B22] Kahana M. Loftus G. (1999). “Response time versus accuracy in human memory,” in *The Nature of Cognition*, ed. Sternberg R. J. (Cambridge, MA: MIT Press), 322–384.

[B23] Kiefer P. Giannopoulos I. Raubal M. Duchowski A. (2017). Eye tracking for spatial research: cognition, computation, challenges. *Spat. Cogn. Comput.* 17 1–19. 10.1080/13875868.2016.1254634

[B24] Kirchberg V. Tröndle M. (2012). Experiencing exhibitions: a review of studies on visitor experiences in museums. *Curator* 55 435–452. 10.1111/j.2151-6952.2012.00167.x

[B25] Krukar J. (2014). Walk, look, remember: the influence of the gallery’s spatial layout on human memory for an art exhibition. *Behav. Sci.* 4 181–201. 10.3390/bs4030181 25379276 PMC4219265

[B26] Krukar J. Conroy Dalton R. (2013). “Spatial predictors of eye movement in a gallery setting,” in *Eye Tracking for Spatial Research, Proceedings of the 1st International Workshop (in conjunction with COSIT 2013)*, Scarborough, 14–19.

[B27] Leder H. Belke B. Oeberst A. Augustin D. (2004). A model of aesthetic appreciation and aesthetic judgments. *Br. J. Psychol.* 95 489–508. 10.1348/0007126042369811 15527534

[B28] Lefcheck J. S. (2016). PiecewiseSEM: piecewise structural equation modelling in r for ecology, evolution, and systematics. *Methods Ecol. Evol.* 7 573–579. 10.1111/2041-210X.12512

[B29] Locher P. Krupinski E. Mello-Thoms C. Nodine C. (2007). Visual interest in pictorial art during an aesthetic experience. *Spat. Vis.* 21 55–77. 10.1163/156856807782753868 18073051

[B30] Lu Y. Peponis J. (2014). Exhibition visitors are sensitive to patterns of display covisibility. *Environ. Plan. B Plan. Design* 41 53–68. 10.1068/b39058

[B31] Lüdecke D. (2020). *sjPlot: Data Visualization for Statistics in Social Science. R Package Version 2.8.2.* Available at: https://CRAN.R-project.org/package=sjPlot (accessed January 1, 2020).

[B32] Mathôt S. Schreij D. Theeuwes J. (2012). OpenSesame: an open-source, graphical experiment builder for the social sciences. *Behav. Res. Methods* 44 314–324. 10.3758/s13428-011-0168-7 22083660 PMC3356517

[B33] Melton A. W. (1935). *Problems of Installation in Museums of Art.* Washington, DC: American Association of Museums.

[B34] Melton A. W. (1972). Visitor behavior in museums: some early research in environmental design. *Hum. Fact.* 14 393–403. 10.1177/001872087201400503

[B35] Nadal M. Skov M. (2017). Top–down and bottom–up: front to back. *Phys. Life Rev.* 21 148–149. 10.1016/j.plrev.2017.06.013 28622986

[B36] Newhouse V. (2005). *Art and the Power of Placement.* New York, NY: The Monacelli Press.

[B37] Pallasmaa J. (2013). “Museum as an embodied experience,” in *The Multisensory Museum: Cross-Disciplinary Perspectives on Touch, Sound, Smell, Memory, and Space*, eds Levent N. Pascual-Leone A. (Lanham: Rowman & Littlefield Publishers), 239–249.

[B38] Peponis J. Dalton R. C. Wineman J. Dalton N. (2004). Measuring the effects of layout upon visitors’ spatial behaviors in open plan exhibition settings. *Environ. Plan. B Plan. Design* 31 453–473. 10.1068/b3041

[B39] Robinson E. S. (1928). *The Behaviour of the Museum Visitor.* Washington, DC: American Association of Museums.

[B40] Serrell B. (1997). Paying attention: the duration and allocation of visitors’ time in museum exhibitions. *Curator* 40 108–125. 10.1111/j.2151-6952.1997.tb01292.x

[B41] Shipley B. (2013). The AIC model selection method applied to path analytic models compared using a d-separation test. *Ecology* 94 560–564. 10.1890/12-0976.1 23687881

[B42] Smith J. K. Smith L. F. (2001). Spending time on art. *Empiri. Stud. Arts* 19 229–236. 10.2190/5MQM-59JH-X21R-JN5J

[B43] Smith L. F. Smith J. K. Tinio P. P. L. (2017). Time spent viewing art and reading labels. *Psychol. Aesthet. Creat. Arts* 11 77–85. 10.1037/aca0000049

[B44] Stavroulaki G. Peponis J. (2003). “The spatial construction of seeing at Castelvecchio,” in *Proceedings of the 4th International Space Syntax*, (London: UCL).

[B45] Tatler B. W. Gilchrist I. D. Land M. F. (2005). Visual memory for objects in natural scenes: from fixations to object files. *Q. J. Exp. Psychol. A* 58 931–960. 10.1080/02724980443000430 16194942

[B46] Tatler B. W. Land M. F. (2015). “Everyday visual attention,” in *The Handbook of Attention*, eds Fawcett J. Kingstone A. Risko E. (Cambridge, MA: MIT Press).

[B47] Tröndle M. Greenwood S. Bitterli K. van den Berg K. (2014). The effects of curatorial arrangements. *Museum Manag. Curatorsh.* 29 140–173. 10.1080/09647775.2014.888820

[B48] Varoudis T. (2012). *DepthmapX Multi-Platform Spatial Network Analysis Software.* London: The Bartlett School of Architecture.

[B49] von Stülpnagel R. Frankenstein J. (2015). Configurational salience of landmarks: an analysis of sketch maps using space Syntax. *Cogn. Process.* 16 437–441. 10.1007/s10339-015-0726-5 26239756

[B50] Wessel D. Mayr E. Knipfer K. (2007). “Re-viewing the museum visitor’s view,” in *Proceedings of the Workshop Research Methods in Informal and Mobile Learning*, (London: Institute of Education), 17–23.

[B51] Westat F. J. (2010). *The 2010 User-friendly Handbook for Project Evaluation.* Alexandria: National Science Foundation.

[B52] Wineman J. D. Peponis J. (2010). Constructing spatial meaning. *Environ. Behav.* 42 86–109. 10.1177/0013916509335534

[B53] Yalowitz S. S. Bronnenkant K. (2009). Timing and tracking: unlocking visitor behavior. *Visit. Stud.* 12 47–64. 10.1080/10645570902769134

[B54] Yoshimura Y. Girardin F. Carrascal J. P. Ratti C. Blat J. (2012). “New tools for studying visitor behaviours in museums: a case study at the louvre,” in *Information and Communication Technologies in Tourism 2012*, eds Fuchs M. Ricci F. Cantoni L. (Vienna: Springer Vienna), 391–402. 10.1007/978-3-7091-1142-0_34

[B55] Zamani P. (2009). “Architecture as curatorial device,” in *Proceedings of the 7th International Space Syntax Symposium*, eds Koch D. Marcus L. Steen J. (Stockholm: KTH), 1–12.

[B56] Zisch F. Gage S. Spiers H. (2013). “Navigating the museum,” in *The Multisensory Museum: Cross-Disciplinary Perspectives on Touch, Sound, Smell, Memory, and Space*, eds Levent N. Pascual-Leone A. (Lanham: Rowman & Littlefield Publishers), 215–237.

